# Edoxaban treatment in atrial fibrillation in routine clinical care: One‐year outcomes of the prospective observational ETNA‐AF study in South Korean patients

**DOI:** 10.1002/joa3.12878

**Published:** 2023-05-31

**Authors:** Eue‐Keun Choi, Jong‐Il Choi, Hyoung‐Seob Park, Gyo‐Seung Hwang, Boyoung Joung, Jong‐Youn Kim, Dae‐Hyeok Kim, Dong Gu Shin, Hyung Wook Park

**Affiliations:** ^1^ Department of Internal Medicine Seoul National University College of Medicine, Seoul National, University Hospital Seoul South Korea; ^2^ Department of Internal Medicine Korea University College of Medicine, Korea University Anam Hospital Seoul South Korea; ^3^ Department of Internal Medicine Keimyung University College of Medicine, Cardiovascular Center, Keimyung University Dongsan Hospital Daegu South Korea; ^4^ Department of Internal Medicine Ajou University School of Medicine, Ajou University Hospital Suwon South Korea; ^5^ Department of Internal Medicine Yonsei University College of Medicine, Severance Hospital Seoul South Korea; ^6^ Department of Internal Medicine Yonsei University College of Medicine, Gangnam Severance Hospital Seoul South Korea; ^7^ Department of Internal Medicine Inha University College of Medicine, Inha University Hospital Incheon South Korea; ^8^ Department of Internal Medicine Yeungnam University College of Medicine, Yeungnam University Medical Center Daegu South Korea; ^9^ Department of Internal Medicine Chonnam National University School of Medicine, Chonnam National University Hospital Gwangju South Korea

**Keywords:** major bleeding, non‐vitamin K antagonist oral anticoagulants, real‐world, registry, stroke prevention

## Abstract

**Background:**

The real‐world outcomes of edoxaban treatment in patients with atrial fibrillation (AF) were analyzed in the ETNA‐AF (Edoxaban Treatment in Routine Clinical Practice) study involving data from multiple regional registries. This report addresses effectiveness and safety of edoxaban in the Korean ETNA‐AF population.

**Methods:**

One‐year data from 1887 Korean ETNA‐AF participants were analyzed according to edoxaban dose and patient age and compared with results of other ETNA‐AF registries.

**Results:**

Approximately 70% of patients received the recommended doses of edoxaban (60 mg/30 mg); non‐recommended 60 mg and 30 mg doses were prescribed to 9.6% and 19.8% of the patients, respectively. The proportions of reference age (<65 years), youngest‐old (65–74 years) and middle‐old/oldest‐old (≥75 years) groups were 21.4%, 40.2%, and 38.4%, respectively. Incidence of major or clinically relevant nonmajor bleeding was similar within dose (0.57%–1.71%) and age subgroups (1.26%–1.63%). Incidence of net clinical outcome, a composite of stroke, systemic embolic event, major bleeding, and all‐cause mortality, was also comparable among dose subgroups (1.14%–3.10%) and age subgroups (2.28%–2.78%). The percentage of Korean patients receiving non‐recommended 30 mg (19.8%) was over twice that of the European population (8.4%). However, the clinical outcomes were generally similar among different populations included in the ETNA‐AF study.

**Conclusions:**

The outcomes in the Korean ETNA‐AF population are like those in the global ETNA‐AF population, with overall low event rates of stroke, major bleeding and all‐cause mortality across age and dose subgroups. Edoxaban can be used effectively and safely in specific populations of Korean AF patients, including the elderly.

## 1 INTRODUCTION

Atrial fibrillation (AF) is the most common cardiac arrhythmia, with 1%–4% of the general population being affected.^1^, ^2^, ^3^, ^4^ The prevalence of AF in South Korea has been increasing over the last two decades, with a 1.7‐fold increase in the incidence observed between 2008 and 2015.^5^ AF poses multiple risks, with the two most graves being a two‐fold increase in mortality risk and a five‐fold increase in the risk of ischemic stroke when compared with persons without AF.^6^, ^7^ Further, hospitalizations due to AF and its complications, such as aggravation of heart failure, thromboembolic complications, and acute arrhythmia management, constitute a significant burden for healthcare systems, and this form of arrhythmia has a detrimental effect on the quality of life and exercise tolerance of the patients.^8^, ^9^

Many of the risks mentioned above, particularly stroke and venous thromboembolism, can be effectively prevented with anticoagulation therapy. Until 2009, the only oral anticoagulants available for patients with AF were warfarin and vitamin K antagonists (VKAs).^10^ While effective, those drugs have a serious drawback in the form of a narrow therapeutic index which necessitates constant monitoring and dose adjustment. Due to resultant inconvenience, patient adherence to warfarin/VKA treatment tends to be poor, which rather contributes to increasing the risk of bleeding and thromboembolic events.^11^, ^12^, ^13^, ^14^ Most of the drawbacks mentioned above were overcome by new non‐VKA oral anticoagulants (NOACs), including edoxaban. Unlike VKAs, NOACs have a rapid onset and offset of action and, due to their predictable anticoagulant effects, independent of dietary intake of vitamin K, can be administered at fixed doses with no need for routine coagulation monitoring.^15^, ^16^, ^17^, ^18^, ^19^ NOACs are being used more frequently to prevent stroke in patients with AF because of their ease of administration and comparative efficacy compared with warfarin in reducing thromboembolism and major bleeding.^20^

Edoxaban, one of the NOACs, was approved in South Korea and many other countries for the prevention of stroke and systemic embolic event (SEE) in patients with nonvalvular AF. It was also approved to treat deep vein thrombosis and pulmonary embolism.^21^ The approval was based on the results of the ENGAGE AF‐TIMI 48 (Effective Anticoagulation with Factor Xa Next Generation in Atrial Fibrillation–Thrombolysis in Myocardial Infarction 48) and Hokusai‐venous thromboembolism (VTE) randomized controlled clinical trial. In the ENGAGE AF‐TIMI 48 trial, once‐daily edoxaban showed similar efficacy as well managed warfarin in the prevention of stroke or SEE, with significantly lower rates of bleeding (e.g., for major bleeding, hazard ratio [HR] = 0.80_[edoxaban 60 mg/warfarin]_, *p* < .001; HR = 0.47_[edoxaban 30 mg/warfarin]_, *p* < .001) and cardiovascular (CV) mortality (HR = 0.86_[edoxaban 60 mg/warfarin]_, *p* = .01; HR = 0.85_[edoxaban 30 mg/warfarin]_, *p* = .008).^22^

Edoxaban Treatment in Routine Clinical Practice for Patients With Nonvalvular Atrial Fibrillation (ETNA‐AF) was designed as a noninterventional study to collect data from edoxaban‐treated patients with AF from multiple regional registries.^23^, ^24^, ^25^ The results for Korean patients participating in the study were presented elsewhere.^26^, ^27^ Although the safety and effectiveness of edoxaban in routine clinical practice were reported for Korean and Taiwanese AF patients through the ETNA‐AF registry, the data associated with the Korean special populations is limited. The aim of the present report, also based on the results of the ETNA‐AF study, is to address some specific aspects of edoxaban treatment in Asians, especially the South Korean population, such as the effectiveness and safety of the drug according to its dose and patient age. Additionally, the outcomes of edoxaban treatment and baseline characteristics of Korean patients were compared with those of the other regional countries in the ETNA‐AF registry to observe the ethnic difference.

## 2 METHODS

### 2.1 Study design

This multicenter, prospective, noninterventional study (ETNA‐AF‐KOR‐TWN; NCT02951039) was conducted in patients treated with edoxaban for AF both in Korea and Taiwan as a regional study under the global scheme of the ETNA program. The ETNA program was designed to integrate the safety and effectiveness data of edoxaban indicated for AF or VTE in routine practice conditions across Europe, Japan, and East and Southeast Asia. The detailed study design and rationale of the Global ETNA program were previously published,^23^, ^24^ and the overall plan of the ETNA‐AF‐KOR‐TWN stayed the same. Here, we present the study focused on the Korean registry. Two‐thousand patients were to be enrolled under different care settings (primary or secondary care or other specialties) in Korea, and each patient was to be followed up for 2 years. Here, we report a 1‐year follow‐up. To adhere to the noninterventional nature of the study, all clinical procedures and decisions, including modification of edoxaban dose, continuation or discontinuation of edoxaban treatment, and concomitant treatment, were solely based on the physician's clinical judgement for the best interest of the patient.

### 2.2 Target patients

Unselected patients with nonvalvular AF who were treated with edoxaban for a reduction in the risk of stroke and systemic embolism according to the approved local label were eligible to participate in the study if they provided written informed consent. The only exclusion criterion was simultaneously participating in an interventional study. Patients meeting the eligibility criteria were to be consecutively enrolled in each center to the extent possible.

### 2.3 Study variables and outcomes

At baseline, patient demographics and clinical characteristics, laboratory parameters, medical history, and details of edoxaban therapy were collected. Follow‐up data were collected approximately 12 and 24 months after baseline. If a patient's edoxaban treatment was permanently discontinued, the patient was followed up for one more year after the discontinuation of edoxaban or until the end of the 2‐year follow‐up period, whichever came first. As safety outcomes, bleeding events such as major and clinically relevant nonmajor (CRNM) bleeding are presented. For effectiveness outcomes, strokes, systemic embolic events, myocardial infarction, CV mortality, and all‐cause mortality are included. Net clinical outcome is a composite of ischemic or hemorrhagic stroke, SEE, major bleeding, and all‐cause mortality.

### 2.4 Statistical analysis

All analyses were exploratory and descriptive. Only events with a start date later than the date of baseline and before the date of baseline +365 days were included. Continuous variables were summarized as mean ± standard deviation (SD) and categorical variables as frequencies (percentages). The main safety and effectiveness outcomes were presented as annualized event rates (% per year) with 95% confidence intervals (CIs). All results were presented for subgroups based on age and edoxaban dose. Age subgroups were divided into reference (<65 years), youngest‐old (65–74 years), and middle/oldest‐old (≥75 years) groups. For major outcomes, HR of the two older groups relative to the reference age group were presented with 95% CI. Dose subgroups were divided into patients who met none of the dose‐reduction criteria(recommended 60 mg, non‐recommended 30 mg) and patients who met ≥1 of the dose‐reduction criteria (non‐recommended 60 mg, recommended 30 mg) groups. For major outcomes, HR between recommended groups and non‐recommended groups were presented with 95% CI. Event rates were presented alongside those reported in other registries of the ETNA‐AF program and ENGAGE AF‐TIMI 48 study.

### 2.5 Ethical standards

The study was conducted in compliance with the Declaration of Helsinki and Guidelines for Good Pharmacoepidemiology Practice. The study was approved by the institutional review boards for all participating centers before study initiation. All patients provided written informed consent.

## 3 RESULTS

### 3.1 Patient characteristics

**FIGURE 1:**
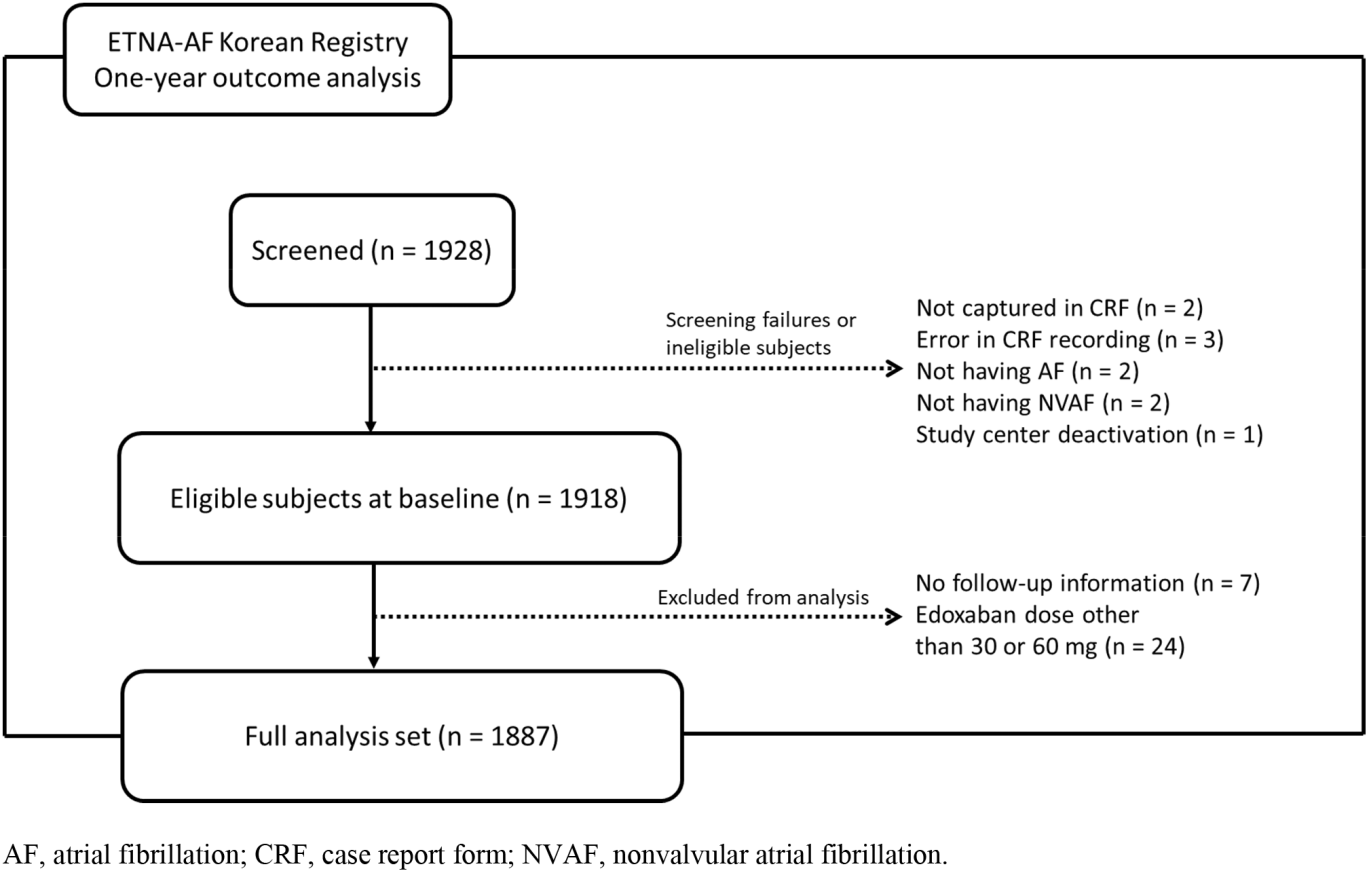
Study cohort diagram. AF, atrial fibrillation; CRF, case report form; NVAF, nonvalvular atrial fibrillation.

During the period between February 2017 and April 2018, a total of 1928 patients were registered in the study from 37 clinics and hospitals in Korea. For the 1‐year outcome analysis, the data were cut off on 26 October 2020, and 1887 patients were included in the analysis (Figure 1). Of these, 60.7% were male, and the mean ± SD age was 70.9 ± 9.0 years. The proportions of reference age (<65 years), youngest‐old (65–74 years) and middle‐old/oldest‐old (≥75 years) groups were 21.4%, 40.2%, and 38.4%, respectively. Mean CHA_2_DS_2_‐VASc (Congestive heart failure, Hypertension, Age ≥ 75 [doubled], Diabetes, Stroke [doubled], Vascular disease, Age 65–74 years, and Sex category [female]) and HAS‐BLED (Hypertension, Abnormal renal/liver function, Stroke, Bleeding history or predisposition, Labile international normalized ratio, Elderly, Drugs/alcohol concomitantly) bleeding scores and proportions of permanent AF, hypertension, and chronic obstructive pulmonary disease had increasing trends with older age (Table S1). Meanwhile, the proportion of male patients, mean body weight, mean body mass index, and creatinine clearance (CrCl) tended to decrease with age. No patients had CrCl less than 15 mL/min, for which edoxaban is contraindicated in Korea. Lower body weight coupled with decreased renal function (CrCl ≤ 50 mL/min) contributed to a higher frequency of prescribing the reduced dose (30 mg) of edoxaban in older age groups (23.3% [<65 years] vs. 42.2% [65–74 years] or 69.8% [≥75 years]).

### 3.2 Exposure to edoxaban

Around 70% of patients received the recommended dose of edoxaban (41.7% received 60 mg and 28.9% received 30 mg; Table 1). Meanwhile, 9.6% of the patients received non‐recommended 60 mg, and 19.8% received non‐recommended 30 mg. Compared with the recommended 60‐mg group, the non‐recommended 30‐mg group was older (mean 71.8 years vs. 66.7 years), had lower body weight (mean 71.8 kg vs. 72.9 kg) and CrCl (mean 74.6 mL/min vs. 78.9 mL/min), had higher CHA_2_DS_2_‐VASc (mean 3.1 vs. 2.5) and HAS‐BLED (mean 2.3 vs. 2.1) scores, and had higher proportions of patients with hypertension (74.8% vs. 68.4%), valvular disease (7.2% vs. 2.7%; Table S2). Compared with the recommended 30‐mg group, the non‐recommended 60‐mg group was younger (mean 71.5 years vs. 76.1 years) with lower CHA_2_DS_2_‐VASc (mean 3.2 vs. 3.7) score, had higher body weight (mean 58.8 kg vs. 72.9 kg) and CrCl (mean 55.9 mL/min vs. 48.5 mL/min), and had a lower proportion of patients with heart failure (6.6% vs. 14.3%).

**TABLE 1:** Patient demographics and baseline characteristics in overall population.

	Overall (*n* = 1887)
Sex, male	1145 (60.7)
Age, years, mean ± SD	70.9 ± 9.0
<65	404 (21.4)
65–74	759 (40.2)
75–84	645 (34.2)
≥85	79 (4.2)
Weight, kg, mean ± SD	65.8 ± 11.7
Body mass index, kg/m^2^, mean ± SD	24.8 ± 3.5
CrCl, mL/min^a^, mean ± SD	65.8 ± 23.0
≥80	392 (24.4)
50–80	798 (49.7)
30–50	367 (22.9)
15–30	48 (3.0)
CHA_2_DS_2_‐VASc, mean ± SD	3.0 ± 1.4
HAS‐BLED^b^, mean ± SD	2.2 ± 1.0
Type of AF	
Paroxysmal	658 (34.9)
Persistent	42 (28.7)
Long‐standing persistent	319 (16.9)
Permanent	368 (19.5)
Diabetes mellitus	534 (28.3)
Hypertension	335 (70.7)
Heart failure (derived)	182 (9.6)
COPD	74 (3.9)
Peripheral artery disease	6 (0.3)
History of ischemic stroke	326 (17.3)
History of major or CRNM bleeding	43 (2.3)
History of major bleeding	37 (2.0)
Valvular disease	0 (4.2)
Edoxaban dose at baseline	
60 mg	968 (51.3)
Recommended	787 (41.7)
Non‐recommended	181 (9.6)
30 mg	919 (48.7)
Recommended	546 (28.9)
Non‐recommended	373 (19.8)

*Note*: Data are mean ± SD for continuous variables and number (percentage) for categorical variables.

Abbreviations: AF, atrial fibrillation; CHA_2_DS_2_‐VASc, congestive heart failure, hypertension, age (≥75), diabetes, previous stroke/transient ischemic attack, vascular disease, age (65–74), sex (female); COPD, chronic obstructive pulmonary disease; CrCl, creatinine clearance; CRNM, clinically relevant nonmajor; HAS‐BLED, hypertension, abnormal liver/renal function, stroke history, bleeding history or predisposition, elderly, drug/alcohol use; NA, not available; SD, standard deviation.

^a^ Calculated using the Cockcroft‐Gault equation. Percentage calculation was done amongst patients with available data.

^b^ Modified HAS‐BLED excluding labile international normalized ratio.

### 3.3 One‐year outcome by age group

**FIGURE 2:**
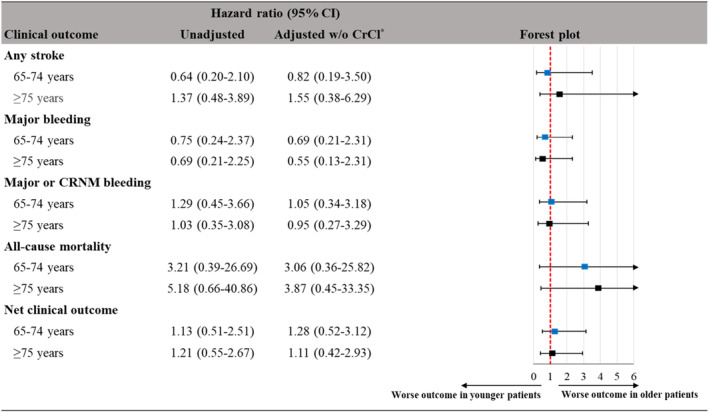
Risk of clinical outcomes in age subgroups (<65 years, reference). *Cox model adjusted for dose (60 mg/30 mg), sex, BMI, CHA_2_DS_2_‐VASc, HAS‐BLED, Type of AF. CrCl is exempted from an additional adjusting factor because collinearity issue occurs due to strong correlation between age and CrCl (*r* = −.66, *p* < .0001).

In terms of effectiveness and safety outcomes, no statistically significant trend was observed across the age subgroups (Figure 2; Table S3). The incidence rate of any stroke in the reference age group (<65 years) at 1 year was 1.26% (95% CI: 0.53%, 3.04%), which was not significantly different from the results of the two older age groups (aHR_[65–74/<65]_ = 0.82, 95% CI: 0.19, 3.50; aHR_[≥75/<65]_ = 1.55, 95% CI: 0.38, 6.29). No systemic embolic event was reported in any of the age groups. For myocardial infarction, no event was captured in the reference age group, whereas one event was recorded in both older age groups. The annual incidence rate of major bleeding in the reference age group was 1.26% (95% CI: 0.53%, 3.04%), which was not significantly different from the results of the two older age groups (aHR_[65–74/<65]_ = 0.69, 95% CI: 0.21, 2.31; aHR_[≥75/<65]_ = 0.55, 95% CI: 0.13, 2.31). Even if CRNM bleeding, which did not occur in the reference age group, was counted together with major bleeding, the incidence rates of the two older age groups were not statistically different from the reference group (aHR_[65–74/<65]_ = 1.05, 95% CI: 0.34, 3.18; aHR_[≥75/<65]_ = 0.95, 95% CI: 0.27, 3.29). The crude annual all‐cause mortality rate increased with age (0.25%, 0.81%, and 1.30% in <65 years, 65–74 years, and ≥75 years, respectively); however, no statistically significant difference was found between the age groups. No statistically significant difference was found either in the incidence of net clinical outcome, a composite of ischemic or hemorrhagic stroke, SEE, major bleeding, and all‐cause mortality, between the reference group (2.28%) and the older age groups (aHR_[65–74/<65]_ = 1.28, 95% CI: 0.52, 3.12; HR_[≥75/<65]_ = 1.11, 95% CI: 0.42, 2.93). No difference between the age groups was detected in the Kaplan–Meier curves in terms of cumulative risk of net clinical outcome up to 1 year (Figure S1).

### 3.4 One‐year outcome by dose subgroup

The annual incidence rates of major clinical outcomes are summarized in Table S4. The annual incidence of any stroke among the dose subgroups ranged from 1.05% (recommended 60 mg) to 1.55% (recommended 30 mg). Myocardial infarction occurred only in the non‐recommended 30‐mg group at the rate of 0.55% (95% CI: 0.14%, 2.20%). Regarding safety outcomes, the non‐recommended 60‐mg group did not experience any safety events except CRNM bleeding in one patient (0.57%). Incidence of major bleeding in the rest of the groups ranged from 0.77% to 1.31%. Incidence of the net clinical outcome was lowest (1.14%) in the non‐recommended 60‐mg group compared with the results of the rest of the dose subgroups, which ranged from 2.51% (recommended 60 mg) to 3.10% (recommended 30 mg).

### 3.5 Comparison with the data from other regions

Patient demographics and other baseline characteristics are presented in Table S5 according to the region studied under the ETNA‐AF program, along with those of the Asian subgroup of the ENGAGE AF‐TIMI 48 study.^28^ When compared with the data from the European registry of the ETNA‐AF program, the characteristics of the Korean patients were mostly comparable, except for lighter body weight (65.8 kg vs. 81.0 kg) and lower CrCl (65.8 mL/min vs. 74.3 mL/min) than European patients, which resulted in the more frequent prescription of 30 mg edoxaban in Korea than in Europe (48.7% vs. 23.6%). Japanese patients had a slightly lower mean body weight than Korean patients (65.8 kg vs. 60.0 kg) and a higher proportion of heart failure history (9.6% vs. 27.1%); otherwise, both regions shared similar patient characteristics. Nonetheless, it should be noted that 72.4% of Japanese patients were treated with 30 mg edoxaban. Also of note, a higher percentage of patients received non‐recommended 30 mg in the Asian population, specifically Korean (19.8%) and Japanese (12.6%) patients, than in the European population (8.4%). Compared with the patient characteristics of the current Korean registry, the Asian subgroups from the ENGAGE AF‐TIMI 48 study had higher CHA_2_DS_2_‐VASc risk scores and HAS‐BLED bleeding risk scores and greater proportions of patients with medical history such as hypertension (70.7% vs. 83.9%) or heart failure (9.6% vs. 50.6%).^28^

**FIGURE 3:**
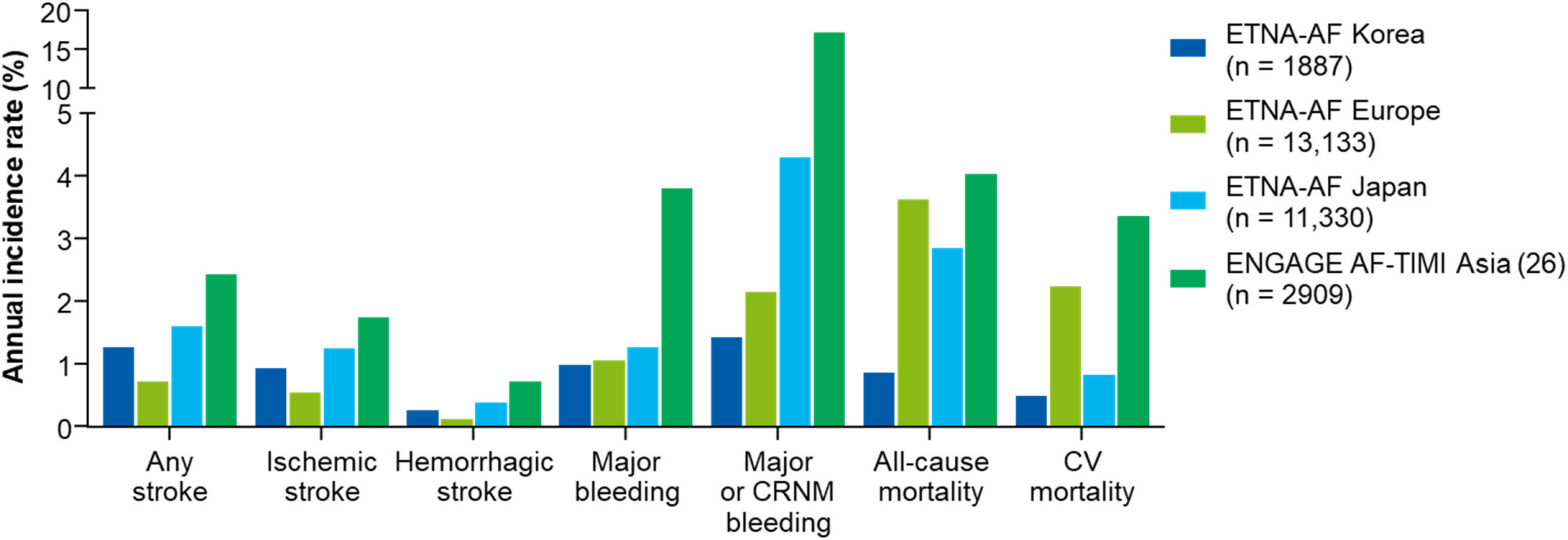
Annual incidence rates of primary clinical outcomes in different populations of ETNA‐AF studies and in ENGAGE AF‐TIMI Asia. CRNM, clinically relevant nonmajor; CV, cardiovascular.

Figure 3 shows annual incidence rates of major clinical outcomes according to the different populations. The annual incidence rate of any stroke in Korea (1.27%) was comparable with that of Japan (1.61%) and the Asian subgroup of the ENGAGE AF‐TIMI 48 study (2.43%), whereas it was higher than that of Europe (0.72%).^28^ Annual incidence rates of major bleeding (0.99%–1.27%) and major or CRNM bleeding (1.43%–4.30%) in the registries of the ETNA‐AF program were similar between the different regions and markedly lower than those observed in the Asian subgroup of the ENGAGE AF‐TIMI 48 study (major bleeding, 3.80%; major or CRNM bleeding, 17.20%).^28^ Both the all‐cause mortality rate (0.87%) and CV mortality (0.49%) rate in Korea were much lower than in Europe (3.62% for all‐cause mortality and 2.24% for CV mortality) and Japan (2.85% for all‐cause mortality and 0.83% for CV mortality). The proportion of the middle/oldest population (≥75 years) in Korea was 38.4%, which was similar to that of the ENGAGE AF‐TIMI 48 study (40.2%) and the data from the East‐Asian registry (41.2%) but was less than the global registry (50.4%).^29^ Patient demographics, baseline characteristics, and percentage of patients treated with 30 mg edoxaban in the middle/oldest population of the Korean registry were similar to those of the East‐Asian registry (Table S6). Compared with the middle/oldest population in ENGAGE AF‐TIMI 48 study, the same age group in the Korean registry had a similar mean age (79.5 years [Korean] vs. 79.4 years [ENGAGE AF‐TIMI 48]) and sex distribution (male proportion, 53.9% vs. 55.4%), lower mean body weight (61.7 kg vs. 77.2 kg), a higher percentage of patients with CrCl ≤50 mL/min (49.2% vs. 36.4%), a lower percentage of patients with permanent AF (24.7% vs. 52.1%), and a lower percentage of patients with hypertension (73.6% vs. 93.7%) and heart failure (10.4% vs. 45.0%).^29^ The percentage of patients treated with 30 mg edoxaban in the middle/oldest population was higher in Korea than in the ENGAGE AF‐TIMI 48 (69.8% vs. 32.9%).

Annual incidence rates of any stroke in the middle/oldest‐old population of ETNA‐AF Global, Korea, and East‐Asia ranged from 1.37% (Global registry) to 1.75% (Korean registry), which was comparable to that of the same age group in the ENGAGE AF‐TIMI 48 study (1.71% for ischemic stroke and 0.35% for hemorrhagic stroke). Major bleeding occurred less frequently in the middle/oldest‐old population of the Korean patients (0.87%, 95% CI: 0.39%, 1.94%) than in the Global (1.49%) or East‐Asian registry (1.90%) or in the ENGAGE AF‐TIMI 48 study (4.68%). All‐cause mortality and CV mortality rates in the middle/oldest‐old population of the Korean registry (1.30% and 0.72%, respectively) were also lower than the outcome from Global or East‐Asia registries (Table S7).

## 4 DISCUSSION

The present analysis, including 1887 Korean patients with AF treated with edoxaban, provided a region‐specific insight into the results of the global real‐world ETNA‐AF study. The results presented herein shed new light on the effectiveness and safety of edoxaban used at non‐recommended doses and, specifically, in more vulnerable populations (patients aged 65–74 years and ≥75 years). Additionally, the study demonstrated differences in the outcomes of edoxaban treatment and other characteristics between Korean patients with AF, patients of other ethnicities included in the ETNA‐AF study,^24^ and Asian participants of the registration trial of edoxaban, ENGAGE AF‐TIMI 48.^22^

Regarding the dosage regimen of edoxaban, approximately 30% of Korean patients participating in the ETNA‐AF study did not receive a recommended dose of the study drug, with non‐recommended 30 mg and non‐recommended 60 mg being prescribed to 19.8% and 9.6% of the patients, respectively. Adherence to treatment and prescription guidelines is a common problem in Asian patients with AF treated with NOACs.^30^, ^31^, ^32^, ^33^ Compared with Western patients, Asian patients are smaller and have a higher risk of bleeding, and therefore, physicians tend to prescribe them lower doses of anticoagulants than the recommended dose.^34^, ^35^, ^36^ The results of the present analysis are consistent with the findings of a Korean registry study, CODE‐AF (COmparison study of Drugs for symptom control and complication prEvention of AF).^33^ In that study, 68% out of 371 patients with AF treated with edoxaban received recommended 60 mg (34.8%) or recommended 30 mg (33.2%), while 23.5% and 8.6% were treated with non‐recommended 30 mg and non‐recommended 60 mg, respectively. Nevertheless, edoxaban showed the highest dose recommendation adherence in that study (recommended dose use 68%), with rivaroxaban having the lowest adherence (recommended dose use 43.9%).^33^ When patients receiving recommended 60 mg of edoxaban were compared with those prescribed non‐recommended 30 mg, the latter group was shown to be characterized by significantly older age, lower body weight, lower CrCl, higher CHA_2_DS_2_‐VASc and HAS‐BLED scores and significantly more frequent history of bleeding events (all, *p* < .001). However, up to 91% of patients prescribed the non‐recommended 30 mg of edoxaban did not meet any of the dose reduction criteria for this agent, and 9% of patients were prescribed a 15‐mg dose of edoxaban.^33^

**FIGURE 4:**
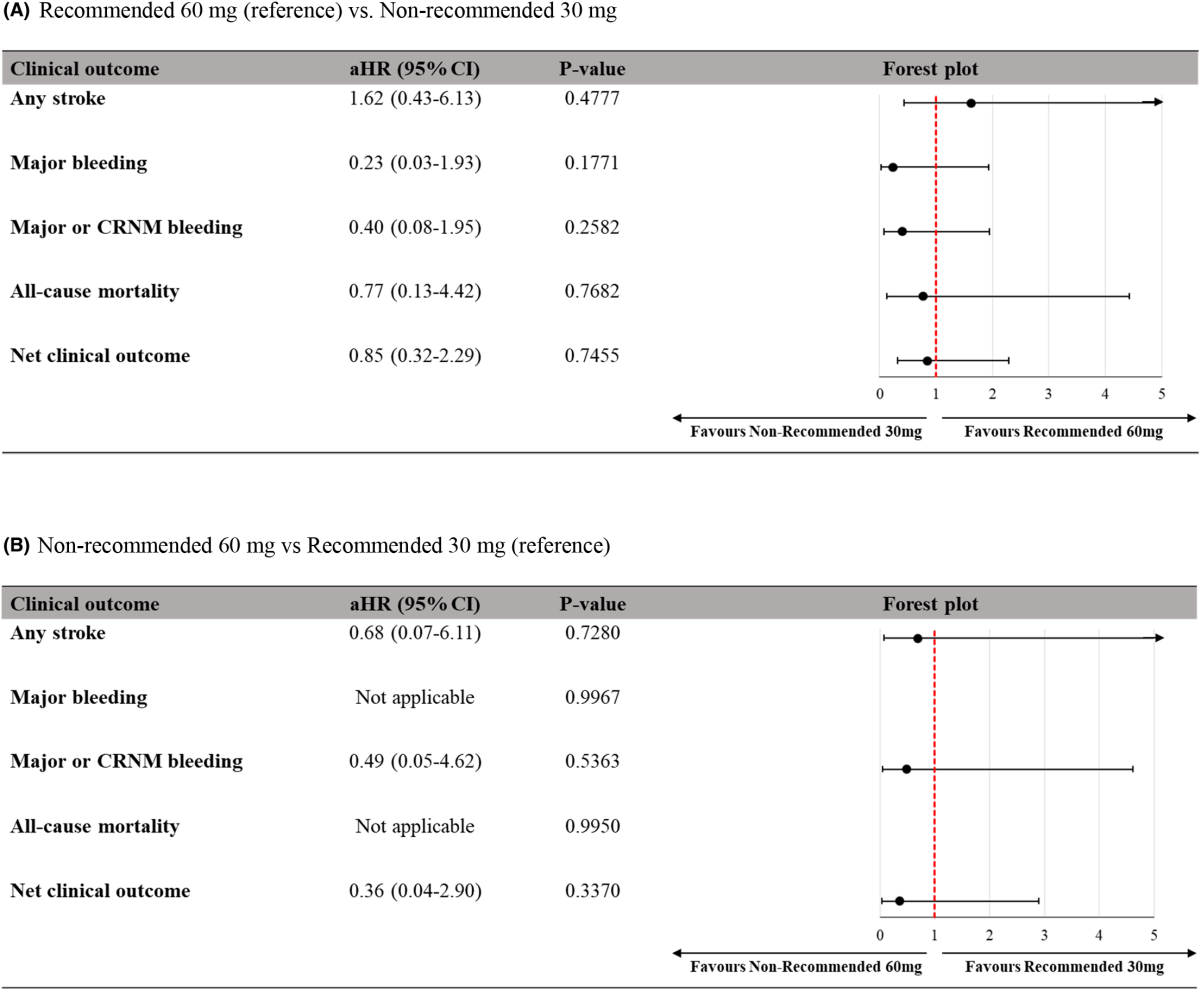
Risk of clinical outcomes in dose subgroups. (A) Recommended 60 mg (reference) vs. Non‐recommended 30 mg. (B) Non‐recommended 60 mg vs. Recommended 30 mg (reference). Data are number of patients (percentage) with 95% confidence interval of the incidence rate in square brackets. aHR, adjusted Hazard Ratio, Cox model adjusted for age, sex, BMI, CrCl, CHA_2_DS_2_‐VASc, HAS‐BLED, Type of AF. CRNM, clinically relevant nonmajor; CV, cardiovascular; and NE, not evaluable.

The evidence from the United States suggests that while the reduction of a NOAC dose improves the safety of anticoagulant treatment by lowering the risk of bleeding, the non‐recommended underdosing is also associated with an increased risk of stroke, which outweighs the safety benefit.^37^, ^38^ Also, the results of a Taiwanese registry study imply that compared with the recommended dosing, underdosing of NOACs (dabigatran, rivaroxaban, apixaban, and edoxaban) was associated with a significantly higher risk of ischemic stroke/systemic embolism (adjusted HR = 1.59, 95% CI: 1.25, 2.02, *p* < .001), whereas overdosing posed an increased risk of major bleeding (adjusted HR = 2.01, 95% CI: 1.13, 3.56, *p* = .017).^30^ Notably, however, on a drug‐specific analysis, non‐recommended dosing of edoxaban was not associated with an increase in stroke/embolism (adjusted HR = 1.43, 95% CI: 0.53, 3.89).^30^ Similarly, the analysis of the European participants of the ETNA‐AF study did not identify differences in the rates of stroke/SEE and ischemic stroke in patients receiving a non‐recommended 30‐mg dose of edoxaban and those treated with the recommended 60 mg.^39^ Also, the results of the present analysis showed that the non‐recommended dosing of edoxaban did not affect much on either the safety or effectiveness of the drug. The annual incidence of any stroke varied from 1.05% in the recommended 60‐mg group to 1.55% in the recommended 30‐mg group, 1.38% in the non‐recommended 30‐mg group and 1.14% in the non‐recommended 60‐mg group without a marked difference between the recommended and non‐recommended dose groups. Also, the dose groups did not differ in terms of the safety outcomes and net clinical outcome. Although major clinical outcomes except for any stroke in the non‐recommended 30‐mg group and all clinical outcomes in the non‐recommended 60‐mg group seem favorable, the statistical power of this study is inadequate in that sample sizes in the non‐recommended dosage groups were small and the event rates were extremely low in real‐world clinical practice (Figure 4). These findings imply that edoxaban represents a safe and effective treatment option also in patients with AF; furthermore, this observation expands the real‐world evidence beyond ENGAGE AF‐TIMI 48. The non‐recommended dosing decision was associated with age, renal function, and history of major bleeding and thrombotic events. However, the approved higher‐dose edoxaban regimen remains the standard therapy among the available edoxaban dosing regimens for stroke prevention in AF. A longer follow‐up and larger sample size are needed to obtain more information about the non‐recommended dosage effects.

The evidence mentioned above seems to be particularly important in the context of administering edoxaban to some vulnerable populations, (e.g., elderly patients). Some previous studies showed that edoxaban and other NOACs might produce clinical benefits in older elderly patients with AF. In a Japanese placebo‐controlled randomized study, a low dose of edoxaban (15 mg) reduced the risk of stroke in ≥80‐year‐old patients with AF who were not eligible for standard anticoagulation treatment.^40^ Also, a Chinese prospective registry study showed that administration of oral anticoagulants, including edoxaban, was an independent predictor of a lower risk of the composite outcome (any thromboembolism, major bleeding and new onset/worsening heart failure) and all‐cause mortality in patients with AF aged ≥85 years.^32^ Those observations are similar to the results of the present analysis in which the incidence of stroke and the annual incidence of major bleeding did not differ significantly among patients aged <65, 65–74, and ≥75 years.

The present analysis highlighted some differences in the outcomes of edoxaban treatment in Korean patients included in the ETNA‐AF study and other participants of this registry study. While Korean patients had a higher annual incidence rate of any stroke than the European participants of the ETNA‐AF study, they presented with lower all‐cause mortality and CV mortality rates; meanwhile, the two populations did not differ substantially in terms of the safety outcomes. These differences mentioned above might be interpreted in the context of the baseline characteristics of the study patients. Korean participants of the ETNA‐AF study tended to be younger than European patients, which may contribute to the differences in the mortality risk. Indeed, older age (both ≥85 and ≥75 years) was identified as a significant contributor to all‐cause mortality in European participants of the ETNA‐AF study.^39^ While the proportion of Korean patients with AF in whom edoxaban was prescribed at 30 mg was substantially higher than the respective percentage of the European participants of the ETNA‐AF study (48.7% vs. 23.6%), in view of the results presented above, this factor was less likely to contribute to a higher incidence of stroke in the Korean registry. It cannot be excluded that the regional differences in the occurrence of stroke, the statistical significance of which was in fact not verified, were instead associated with the disparity of the sample sizes. The overall number of European patients participating in the ETNA‐AF study (*n* = 13133) was substantially higher than the number of Korean participants (*n* = 1887), and hence, the percentages of stroke in the latter group might be well overestimated.

The present study was not free from potential limitations. First, the outcomes of edoxaban treatment were not verified against a comparator, whether a NOAC or a VKA. Second, the study participants were followed for a relatively short time, and notably, the available follow‐up for Korean patients was shorter than for those from Europe. While these limitations should be considered during the interpretation of the results, the strengths of the study related to its real‐world character should also be highlighted; namely, the large sample size and the fact that all therapeutic decisions were solely at physicians' discretion.

## 5 CONCLUSIONS

ETNA‐AF Korea is the first prospective, noninterventional study to investigate the effectiveness and safety of all edoxaban doses in a Korean AF population. The annual incidence rates of stroke, major bleeding and all‐cause mortality were low overall across age and dose subgroups and comparable to those in the Global ETNA population. Our observation confirmed that edoxaban could be used safely and effectively even in the elderly patients and those with ineligible for standard anticoagulation therapy and requiring dose adjustment in routine care in Korea.

## FUNDING INFORMATION

This study was funded by Daiichi Sankyo, Inc.

## CONFLICT OF INTEREST STATEMENT

Eue‐Keun Choi reports research grants from Bayer; BMS/Pfizer; Biosense Webster; Chong Kun Dang; Daiichi Sankyo; Dreamtech Co., Ltd.; Medtronic; Samjinpharm; Sanofi‐Aventis; Skylabs; and Yuhan. Jong‐Il Choi reports honoraria from Daiichi Sankyo, Inc.; Boehringer‐Ingelheim; Abbott; Sanofi Genzyme; Samjin pharma; Yuhan; Chong Keun Dang and research grants from Sanofi Genzyme; Medtronic; Chong Keun Dang. Hyoung‐Seob Park reports no conflicts of interest. Gyo‐Seung Hwang reports no conflicts of interest. Boyoung Joung reports research funds from Medtronic, Boston Sicentifics, Abbott, Samjinpharm, Hanmi and Huino. Jong‐Youn Kim reports no conflicts of interest. Dae‐Hyeok Kim reports no conflicts of interest. Dong Gu Shin reports no conflicts of interest. Hyung Wook Park reports no conflicts of interest.

## DECLARATIONS

*Approval of The Research Protocol*: The study was approved by the institutional review boards for all participating centers before study initiation. *Informed consent*: All participating patients provided informed consent. *Registry and the Registration No. of the Study/Trial*: The study was registered in the Clinical Trial Registry with number NCT02951039. *Animal Studies*: N/A.

## Supporting information


Data S1.

## Data Availability

The data underlying this article cannot be shared publicly, as the Global ETNA‐AF program is currently ongoing.
