# Supplementary material for: Edoxaban treatment in atrial fibrillation in routine clinical care: One‐year outcomes of the prospective observational ETNA‐AF study in South Korean patients
Source: J Arrhythm. 2023 May 31;39(4):546–55. doi: 10.1002/joa3.12878 (PMC10407161; doi:10.1002/joa3.12878)
Supplement: Supplementary file 1 — Data S1. [file JOA3-39-546-s001.docx]

Supplementary Materials

**Supplementary Table 1.** Patient demographics and baseline characteristics in age subgroups

|  | **<65 years (n = 404)** | **65–74 years (n = 759)** | **≥75 years (n = 724)** | ***P*-value** |
| --- | --- | --- | --- | --- |
| **Sex, male** | 277 (68.6) | 478 (63.0) | 390 (53.9) | **<0.0001** |
| **Age, years, mean ± SD** | 57.8 ± 5.7 | 69.7 ± 2.9 | 79.5 ± 3.8 | NA |
| <65 | 404 (100) | 0 (0) | 0 (0) | NA |
| 65–74 | 0 (0) | 759 (100) | 0 (0) |  |
| 75–84 | 0 (0) | 0 (0) | 645 (89.1) |  |
| ≥85 | 0 (0) | 0 (0) | 79 (10.9) |  |
| **Weight, kg, mean ± SD** | 71.9 ± 12.2 | 66.3 ± 10.8 | 61.7 ± 10.7 | **<0.0001** |
| **Body mass index, kg/m^2^, mean ± SD** | 25.7 ± 3.7 | 24.9 ± 3.3 | 24.2 ± 3.5 | **<0.0001** |
| **CrCl, mL/min^a^, mean ± SD** | 87.5 ± 24.4 | 67.8 ± 17.8 | 51.4 ± 15.7 | **<0.0001** |
| ≥80 | 208 (60.3) | 152 (23.2) | 32 (5.3) |  |
| 50–80 | 123 (35.7) | 399 (61.0) | 276 (45.5) |  |
| 30–50 | 13 (3.8) | 98 (15.0) | 256 (42.2) |  |
| 15–30 | 1 (0.3) | 5 (0.8) | 42 (6.9) |  |
| **CHA_2_DS_2_-VASc, mean ± SD** | 1.8 ± 1.1 | 2.8 ± 1.1 | 4.0 ± 1.3 | **<0.0001** |
| **HAS-BLED^b^, mean ± SD** | 1.5 ± 1.0 | 2.4 ± 1.0 | 2.4 ±1.0 | **<0.0001** |
| **Type of AF** |  |  |  | **<0.0001** |
| Paroxysmal | 156 (38.6) | 270 (35.6) | 232 (32.0) |  |
| Persistent | 141 (34.9) | 205 (27.0) | 196 (27.1) |  |
| Long-standing persistent | 52 (12.9) | 150 (19.8) | 117 (16.2) |  |
| Permanent | 55 (13.6) | 134 (17.7) | 179 (24.7) |  |
| **Diabetes mellitus** | 125 (30.9) | 214 (28.2) | 195 (26.9) | 0.1637 |
| **Hypertension** | 261 (64.6) | 541 (71.3) | 533 (73.6) | **0.0022** |
| **Heart failure (derived)** | 43 (10.6) | 64 (8.4) | 75 (10.4) | 0.9065 |
| **COPD** | 6 (1.5) | 28 (3.7) | 40 (5.5) | **0.0007** |
| **Peripheral artery disease** | 0 (0.0) | 3 (0.4) | 3 (0.4) | 0.2825 |
| **History of ischemic stroke** | 70 (17.3) | 112 (14.8) | 144 (19.9) | 0.1308 |
| **History of major or CRNM bleeding** | 11 (2.7) | 8 (1.1) | 24 (3.3) | 0.2431 |
| **History of major bleeding** | 11 (2.7) | 6 (0.8) | 20 (2.8) | 0.5486 |
| **Valvular disease** | 3 (0.7) | 46 (6.1) | 31 (4.3) | **0.0288** |
| **Edoxaban dose at baseline** |  |  |  |  |
| 60 mg | 310 (76.7) | 439 (57.8) | 219 (30.2) |  |
| Recommended | 278 (68.8) | 357 (47.0) | 152 (21.0) |  |
| Non-recommended | 32 (7.9) | 82 (10.8) | 67 (9.3) |  |
| 30 mg | 94 (23.3) | 320 (42.2) | 505 (69.8) | **<0.0001^c^** |
| Recommended | 30 (7.4) | 172 (22.7) | 344 (47.5) | **<0.0001^d^** |
| Non-recommended | 64 (15.8) | 148 (19.5) | 161 (22.2) | **<0.0001^e^** |

Data are mean ± SD for continuous variables and number (percentage) for categorical variables. ^a^Calculated using the Cockcroft-Gault equation. Percentage calculation was done amongst patients with available data. ^b^Modified HAS-BLED excluding labile international normalized ratio. ^c^Recommended 30 mg vs Recommended 60 mg. ^d^Recommended 30 mg vs Non-recommended 60 mg. ^e^Non-recommended 30 mg vs Recommended 60 mg.

AF, atrial fibrillation; CHA_2_DS_2_-VASc, congestive heart failure, hypertension, age (≥75), diabetes, previous stroke/transient ischemic attack, vascular disease, age (65–74), sex (female); COPD, chronic obstructive pulmonary disease; CrCl, creatinine clearance; CRNM, clinically relevant nonmajor; HAS-BLED, hypertension, abnormal liver/renal function, stroke history, bleeding history or predisposition, elderly, drug/alcohol use; NA, not available; SD, standard deviation.

**Supplementary Table 2.** Patient demographics and baseline characteristics in dose subgroups

|  | **60 mg**  **(n = 968)** | **30 mg**  **(n = 919)** | **P-value** | **Recommended 60 mg**  **(n = 787)** | **Non-recommended 30 mg**  **(n = 373)** | **P-value** | **Non-recommended 60 mg (n = 181)** | **Recommended 30 mg  (n = 546)** | **P-value** |
| --- | --- | --- | --- | --- | --- | --- | --- | --- | --- |
| **Sex, male** | 703 (72.6) | 442 (48.1) | **<0.0001** | 614 (78.0) | 249 (66.8) | **<0.0001** | 89 (49.2) | 193 (35.3) | **0.0009** |
| **Age, years, mean ± SD** | 67.6 ± 8.7 | 74.4 ± 8.0 | **<0.0001** | 66.7 ± 8.7 | 71.8 ± 8.4 | **<0.0001** | 71.5 ± 7.3 | 76.1 ± 7.3 | **<0.0001** |
| <65 | 310 (32.0) | 94 (10.2) | **<0.0001** | 278 (35.3) | 64 (17.2) | **<0.0001** | 32 (17.7) | 30 (5.5) | **<0.0001** |
| 65–74 | 439 (45.4) | 320 (34.8) |  | 357 (45.4) | 148 (39.7) |  | 82 (45.3) | 172 (31.5) |  |
| 75–85 | 210 (21.7) | 435 (47.3) |  | 148 (18.8) | 149 (39.9) |  | 62 (34.3) | 286 (52.4) |  |
| ≥85 | 9 (0.9) | 70 (7.6) |  | 4 (0.5) | 12 (3.2) |  | 5 (2.8) | 58 (10.6) |  |
| **Weight, kg, mean ± SD** | 70.0 ± 10.5 | 61.4 ± 11.2 | NA**<0.0001****^c^** | 72.9 ± 9.3 | 71.8 ± 8.7 | **0.0270** | 58.8 ± 6.9 | 55.8 ± 8.0 | **<0.0001** |
| **Body mass index, kg/m^2^, mean ± SD** | 25.5 ± 3.3 | 24.1 ± 3.6 | NA**<0.0001^c^** | 26.2 ± 3.2 | 26.4 ± 3.4 | 0.2767 | 23.1 ± 2.7 | 22.8 ± 3.0 | 0.3343 |
| **CrCl, mL/min^a^, mean ± SD** | 73.9 ± 21.5 | 57.6 ± 21.6 | NA**<0.0001****^c^** | 78.9 ± 20.0 | 74.6 ± 20.5 | **0.0003** | 55.9 ± 16.7 | 48.5 ± 15.9 | **<0.0001** |
| ≥80 | 279 (34.4) | 113 (14.2) | NA**<0.0001^c^** | 266 (42.0) | 93 (33.6) | **0.0164** | 13 (7.3) | 20 (3.9) | **<0.0001** |
| 50–80 | 449 (55.4) | 349 (43.9) |  | 367 (58.0) | 184 (66.4) |  | 82 (46.3) | 165 (31.9) |  |
| 30–50 | 78 (9.6) | 289 (36.4) |  | 0 (0.0) | 0 (0.0) |  | 78 (44.1) | 289 (55.8) |  |
| 15–30 | 4 (0.5) | 44 (5.5) |  | 0 (0.0) | 0 (0.0) |  | 4 (2.3) | 44 (8.5) |  |
| **CHA_2_DS_2_-VASc, mean ± SD** | 2.6 ± 1.4 | 3.5 ± 1.4 | **<0.0001** | 2.5 ± 1.4 | 3.1 ± 1.3 | **<0.0001** | 3.2 ± 1.3 | 3.7 ± 1.3 | **<0.0001** |
| **HAS-BLED^b^, mean ± SD** | 2.1 ± 1.0 | 2.3 ± 1.0 | **<0.0001** | 2.1 ± 1.0 | 2.3 ± 0.9 | **0.0005** | 2.2 ± 1.1 | 2.3 ± 1.0 | 0.1082 |
| **Type of AF** |  |  |  |  |  |  |  |  |  |
| Paroxysmal | 352 (36.4) | 306 (33.3) | 0.0758 | 284 (36.1) | 128 (34.3) | **0.0001** | 68 (37.6) | 178 (32.6) | 0.2332 |
| Persistent | 284 (29.3) | 258 (28.1) |  | 248 (31.5) | 108 (29.0) |  | 36 (19.9) | 150 (27.5) |  |
| Long-standing persistent | 143 (14.8) | 176 (19.2) |  | 113 (14.4) | 91 (24.4) |  | 30 (16.6) | 85 (15.6) |  |
| Permanent | 189 (19.5) | 179 (19.5) |  | 142 (18.0) | 46 (12.3) |  | 47 (26.0) | 133 (24.4) |  |
| **Diabetes mellitus** | 286 (29.5) | 248 (27.0) | 0.2173 | 240 (30.5) | 110 (29.5) | 0.7276 | 46 (25.4) | 138 (25.3) | 0.9701 |
| **Hypertension** | 662 (68.4) | 673 (73.2) | **0.0208** | 538 (68.4) | 279 (74.8) | **0.0248** | 124 (68.5) | 394 (72.2) | 0.3467 |
| **Heart failure (derived)** | 68 (7.0) | 114 (12.4) | **<0.0001** | 56 (7.1) | 36 (9.7) | 0.1355 | 12 (6.6) | 78 (14.3) | **0.0067** |
| **COPD** | 33 (3.4) | 41 (4.5) | 0.2392 | 27 (3.4) | 14 (3.8) | 0.7811 | 6 (3.3) | 27 (4.9) | 0.3612 |
| **Peripheral artery disease** | 4 (0.4) | 2 (0.2) | 0.4506 | 4 (0.5) | 1 (0.3) | 0.5598 | 0 (0.0) | 1 (0.2) | 0.5645 |
| **History of ischemic stroke** | 176 (18.2) | 150 (16.3) | 0.2855 | 134 (17.0) | 56 (15.0) | 0.3868 | 42 (23.2) | 94 (17.2) | 0.0734 |
| **History of major or CRNM bleeding** | 12 (1.2) | 31 (3.4) | **0.0019** | 10 (1.3) | 10 (2.7) | 0.0848 | 2 (1.1) | 21 (3.8) | 0.0679 |
| **History of major bleeding** | 11 (1.1) | 26 (2.8) | **0.0080** | 9 (1.1) | 10 (2.7) | 0.0540 | 2 (1.1) | 16 (2.9) | 0.1708 |
| **Valvular disease** | 25 (2.6) | 55 (6.0) | **0.0002** | 21 (2.7) | 27 (7.2) | **0.0003** | 4 (2.2) | 28 (5.1) | 0.0972 |

Data are mean ± SD for continuous variables and number (percentage) for categorical variables. ^a^Calculated using the Cockcroft-Gault equation. Percentage calculation was done amongst patients with available data. ^b^Modified HAS-BLED excluding labile international normalized ratio. ^c^NA, not applicable, these variables are part of criteria for giving low dose.

AF, atrial fibrillation; CHA_2_DS_2_-VASc, congestive heart failure, hypertension, age (≥75), diabetes, previous stroke/transient ischemic attack, vascular disease, age (65–74), sex (female); COPD, chronic obstructive pulmonary disease; CrCl, creatinine clearance; CRNM, clinically relevant nonmajor; HAS-BLED, hypertension, abnormal liver/renal function, stroke history, bleeding history or predisposition, elderly, drug/alcohol use; SD, standard deviation.

**Supplementary Table 3.** Annual incidence rate (%) of clinical outcomes in age subgroups

|  | Overall | | <65 years (n=404) | | 65-74 years (n=759) | | ≥75 years (n=724) | |
| --- | --- | --- | --- | --- | --- | --- | --- | --- |
|  | **Event** | **Annual incidence rate (95% CI)** | **Event** | **Annual incidence rate (95% CI)** | **Event** | **Annual incidence rate (95% CI)** | **Event** | **Annual incidence rate (95% CI)** |
| Any stroke | 23 | 1.27 (0.84; 1.90) | 5 | 1.26 (0.53; 3.04) | 6 | 0.81 (0.37; 1.81) | 12 | 1.75 (1.00; 3.09) |
| Ischemic stroke | 17 | 0.93 (0.58; 1.50) | 4 | 1.01 (0.38; 2.69) | 6 | 0.81 (0.37; 1.81) | 7 | 1.02 (0.49; 2.14) |
| Hemorrhagic stroke | 5 | 0.27 (0.11; 0.66) | 1 | 0.25 (0.04; 1.79) | 0 | 0.00 (0.00; NE) | 4 | 0.58 (0.22; 1.55) |
| Systemic embolic event | 0 | 0.00 (0.00; NE) | 0 | 0.00 (0.00; NE) | 0 | 0.00 (0.00; NE) | 0 | 0.00 (0.00; NE) |
| Myocardial infarction | 2 | 0.11 (0.03; 0.44) | 0 | 0.00 (0.00; NE) | 1 | 0.13 (0.02; 0.96) | 1 | 0.14 (0.02; 1.03) |
| Major bleeding | 18 | 0.99 (0.62; 1.57) | 5 | 1.26 (0.53; 3.04) | 7 | 0.95 (0.45; 1.99) | 6 | 0.87 (0.39; 1.94) |
| Intracranial hemorrhage | 5 | 0.27 (0.11; 0.66) | 1 | 0.25 (0.04; 1.79) | 0 | 0.00 (0.00; NE) | 4 | 0.58 (0.22; 1.55) |
| Major GI bleeding | 5 | 0.27 (0.11; 0.66) | 2 | 0.50 (0.13; 2.01) | 1 | 0.13 (0.02; 0.96) | 2 | 0.29 (0.07; 1.16) |
| Major or CRNM bleeding | 26 | 1.43 (0.97; 2.10) | 5 | 1.26 (0.53; 3.04) | 12 | 1.63 (0.93; 2.87) | 9 | 1.31 (0.68; 2.52) |
| All-cause mortality | 16 | 0.87 (0.54; 1.43) | 1 | 0.25 (0.04; 1.78) | 6 | 0.81 (0.36; 1.80] | 9 | 1.30 (0.68; 2.50) |
| CV mortality | 9 | 0.49 (0.26; 0.94) | 0 | 0.00 (0.00; NE) | 4 | 0.54 (0.20; 1.43) | 5 | 0.72 (0.30; 1.74) |
| Net clinical outcome | 47 | 2.60 (1.95; 3.46) | 9 | 2.28 (1.19; 4.39) | 19 | 2.60 (1.66; 4.07) | 19 | 2.78 (1.77; 4.36) |

Data presented are number (percentage) and 95% confidence interval in square bracket.

CRNM, clinically relevant non-major; CV, cardiovascular; HR, hazard ratio; NE, not evaluable.

**Supplementary Table 4.** Annual incidence rate (%) of clinical outcomes in dose subgroups

**(A)** Recommended 60 mg (reference) vs. Non-recommended 30 mg

|  | **Recommended 60 mg (n = 787)** | | **Non-recommended 30 mg (n = 373)** | | **Hazard ratio (95% CI)** | |
| --- | --- | --- | --- | --- | --- | --- |
|  | **Event** | **Annual incidence rate (95% CI)** | **Event** | **Annual incidence rate (95% CI)** | **Unadjusted** | **Adjusted****^*^** |
| **Any stroke** | 8 | 1.05 (0.52; 2.10) | 5 | 1.38 (0.58; 3.33) | 1.26 (0.30-5.28) | 1.62 (0.43-6.13) |
| Ischemic stroke | 7 | 0.92 (0.44; 1.93) | 3 | 0.83 (0.27; 2.57) | 0.90 (0.23-3.48) | 1.71 (0.38-7.71) |
| Hemorrhagic stroke | 1 | 0.13 (0.02; 0.92) | 1 | 0.27 (0.04; 1.95) | 2.10 (0.13-33.65) | NA |
| **Systemic embolic event** | 0 | 0.00 | 0 | 0.00 | NA | NA |
| **Myocardial infarction** | 0 | 0.00 (0.00; NE) | 2 | 0.55 (0.14; 2.20) | NA | NA |
| **Major bleeding** | 10 | 1.31 (0.70; 2.43) | 4 | 1.11 (0.41; 2.95) | 0.84 (0.26-2.69) | 0.23 (0.03-1.93) |
| Intracranial hemorrhage | 1 | 0.13 (0.02; 0.92) | 1 | 0.27 (0.04; 1.95) | 1.26 (0.30-5.28) | NA |
| Major GI bleeding | 3 | 0.39 (0.13; 1.21) | 1 | 0.27 (0.04; 1.95) | 0.70 (0.07-6.75) | NA |
| **Major or CRNM bleeding** | 13 | 1.71 (0.99; 2.94) | 5 | 1.38 (0.58; 3.32) | 0.81 (0.29-2.27) | 0.40 (0.08-1.95) |
| **All-cause mortality** | 5 | 0.65 (0.27; 1.56) | 3 | 0.82 (0.27; 2.55) | 1.26 (0.30-5.28) | 0.77 (0.13-4.42) |
| **CV mortality** | 4 | 0.52 (0.20; 1.39) | 1 | 0.27 (0.04; 1.95) | 0.53 (0.06-4.70) | NA |
| **Net clinical outcome** | 19 | 2.51 (1.60; 3.93) | 10 | 2.78 (1.50; 5.17) | 1.11 (0.52-2.39) | 0.85 (0.32-2.29) |

**(B)** Non-recommended 60 mg vs Recommended 30 mg (reference)

|  | **Non-recommended 60 mg (n = 181)** | | **Recommended 30 mg (n = 546)** | | **Hazard ratio (95% CI)** | |
| --- | --- | --- | --- | --- | --- | --- |
|  | **Event** | **Annual incidence rate (95% CI)** | **Event** | **Annual incidence rate (95% CI)** | **Unadjusted** | **Adjusted^*^** |
| **Any stroke** | 2 | 1.14 (0.29; 4.56) | 8 | 1.55 (0.77; 3.09) | 0.74 (0.16-3.49) | 0.68 (0.07-6.11) |
| Ischemic stroke | 2 | 1.14 (0.29; 4.56) | 5 | 0.96 (0.40; 2.31) | 1.19 (0.23-6.11) | 1.34 (0.12-14.56) |
| Hemorrhagic stroke | 0 | 0.00 (0.00; NE) | 3 | 0.58 (0.19; 1.79) | 0.00 (0.00-NE) | 0.00 (0.00-NE) |
| **Systemic embolic event** | 0 | 0.00 | 0 | 0.00 | NA | NA |
| **Myocardial infarction** | 0 | 0.00 (0.00; NE) | 0 | 0.00 (0.00; NE) | NA | NA |
| **Major bleeding** | 0 | 0.00 (0.00; NE) | 4 | 0.77 (0.29; 2.05) | 0.00 (0.00-NE) | 0.00 (0.00-NE) |
| Intracranial hemorrhage | 0 | 0.00 (0.00; NE) | 3 | 0.58 (0.19; 1.79) | 0.00 (0.00-NE) | 0.00 (0.00-NE), |
| Major GI bleeding | 0 | 0.00 (0.00; NE) | 1 | 0.19 (0.03; 1.36) | 0.00 (0.00-NE) | 0.00 (0.00-NE) |
| **Major or CRNM bleeding** | 1 | 0.57 (0.08; 4.04) | 7 | 1.35 (0.65; 2.84) | 0.43 (0.05-3.47) | 0.49 (0.05-4.62) |
| **All-cause mortality** | 0 | 0.00 (0.00; NE) | 8 | 1.53 (0.77; 3.06) | 0.00 (0.00-NE) | 0.00 (0.00-NE) |
| **CV mortality** | 0 | 0.00 (0.00; NE) | 4 | 0.77, (0.29; 2.04) | 0.00 (0.00-NE) | 0.00 (0.00-NE) |
| **Net clinical outcome** | 2 | 1.14 (0.29; 4.56) | 16 | 3.10, (1.90; 5.06) | 0.37 (0.09-1.62) | 0.36 (0.04-2.90) |

Data are number of patients (percentage) with 95% confidence interval of the incidence rate in square brackets.

^*^Cox model adjusted for age, sex, BMI, CrCl, CHA_2_DS_2_-VASc, HAS-BLED, Type of AF.

CRNM, clinically relevant nonmajor; CV, cardiovascular; GI, gastrointestinal; and NE, not evaluable.

**Supplementary Table 5.** Comparison of baseline characteristics between regions

|  | **ETNA-AF Korea**  **(n = 1887)** | **ETNA-AF Europe**  **(n = 13,133)** | **ETNA-AF Japan**  **(n = 11,330)** | **ENGAGE AF-TIMI Asia** (1)  **(n = 2909)** |
| --- | --- | --- | --- | --- |
| **Sex, male** | 1145 (60.7) | 7451 (56.7) | 6728 (59.4) | 1960 (67.4) |
| **Age, years, mean ± SD** | 70.9 ± 9.0 | 73.6 ± 9.5 | 74.2 ± 10.1 | 68.7 ± 9.7 |
| **Body weight, kg, mean ± SD** | 65.8 ± 11.7 | 81.0 ± 17.3 | 60.0 ± 12.8 | 66.4 ± 13.1 |
| **CrCl, mL/min,^a^ mean ± SD** | 65.8 **±** 23.0 | 74.3 **±** 30.4 | 63.9 **±** 25.8 | 63.1 ± 22.4 |
| **CHA_2_DS_2_-VASc, mean ± SD** | 3.0 ± 1.4 | 3.2 ± 1.4 | 3.5 ± 1.6 | 4.1 ± 1.3 |
| **HAS-BLED^b^, mean ± SD** | 2.2 ± 1.0 | 2.5 ± 1.1 | 2.4 ± 1.1 | 2.7 ± 1.1 |
| **Edoxaban dose** |  |  |  |  |
| 60 mg, % | 51.3 | 76.4 | 27.6 | 51.1 |
| Recommended | 787 (41.7) | 8916 (67.9) | 2866 (25.3) |  |
| Non-recommended | 181 (9.6) | 1120 (8.5) | 257 (2.3) |  |
| 30 mg, % | 48.7 | 23.6 | 72.4 | 48.9 |
| Recommended | 546 (28.9) | 1992 (15.2) | 6777 (59.8) |  |
| Non-recommended | 373 (19.8) | 1105 (8.4) | 1430 (12.6) |  |
| **Type of AF** |  |  |  |  |
| Paroxysmal | 658 (34.9) | 7056 (53.8) | 5158 (50.9) | 655 (22.5) |
| Persistent | 542 (28.7) | 3175 (24.2) | 1075 (10.6) | 767 (26.4) |
| Long-standing persistent | 319 (16.9) | 320 (2.4) | 2172 (21.4) | NA |
| Permanent | 368 (19.5) | 2557 (19.5) | 1723 (17.0) | 1485 (51.1) |
| **Diabetes mellitus** | 534 (28.3) | 2885 (22.0) | 2622 (23.1) | 992 (34.1) |
| **Hypertension** | 1335 (70.7) | 10,129 (77.1) | 8096 (71.5) | 2442 (83.9) |
| **Heart failure** | 182 (9.6) | 1854 (14.1) | 3068 (27.1) | 1472 (50.6) |
| **COPD** | 74 (3.9) | 1207 (9.2) | 77 (0.7) |  |
| **Peripheral arterial disease** | 6 (0.3) | 427 (3.3) | 178 (1.6) | 66 (2.3) |
| **History of ischemic stroke** | 326 (17.3) | 787 (6.0) | 2018 (17.8) | 1166 (40.1) |
| **History of major or CRNM bleeding** | 43 (2.3) | 273 (2.1) | 417 (3.7) |  |
| **History of major bleeding** | 37 (2.0) | 136 (1.0) | 286 (2.5) |  |
| **Valvular disease** | 80 (4.2) | 2286 (17.4) | 540 (4.8) | 235 (8.1) |

Data are mean ± SD for continuous variables and number of patients (percentage) for categorical variables, unless otherwise noted. ^a^Calculated using the Cockcroft-Gault equation. Percentage calculation was done amongst patients with available data. ^b^Modified HAS-BLED excluding labile international normalized ratio.

AF, atrial fibrillation; CHA_2_DS_2_-VASc, congestive heart failure, hypertension, age (≥75), diabetes, previous stroke/transient ischemic attack, vascular disease, age (65–74), sex (female); COPD, chronic obstructive pulmonary disease; CrCl, creatinine clearance; CRNM, clinically relevant nonmajor; HAS-BLED, hypertension, abnormal liver/renal function, stroke history, bleeding history or predisposition, elderly, drug/alcohol use; NA, not available; SD, standard deviation.

**Supplementary Table 6.** Patient demographics and baseline characteristics of middle/oldest-old population by regions

|  | **ETNA-AF Korea**  **≥75 years** | **ETNA-AF Global**  **≥75 years** | **ETNA-AF East-Asia**  **≥75 years (2)** | **ENGAGE AF-TIMI 48**  **≥75 years (3)** | **ENGAGE AF-TIMI 48 Asia ≥75 years (3)** |
| --- | --- | --- | --- | --- | --- |
| **Patients** | 724 (38.4) | 13,809 (50.4) | 1385 (41.2) | 8474 (40.2) | 979 (34.1) |
| **Sex, male** | 390 (53.9) | 7078 (51.3) | 735 (53.1) | 4697 (55.4) | 624 (63.7) |
| **Age, years, mean ± SD** | 79.5 ± 3.8 | 81.2 ± 4.6 | 80.6 ± 4.5 | 79.4 ± 3.6 | 78.5 ± 3.2 |
| **Body weight, kg, mean ± SD** | 61.7 ± 10.7 | 66.1 ± 16.1 | 62.0 ± 11.4 | 77.2 ± 16.1 | 62.2 ± 11.1 |
| **CrCl, ml/min^a^, mean ± SD** | 51.4 ± 15.7 | 54.2 ± 18.4 | 48.3 ± 16.5 | 58.1 ± 17.9 | 49.5 ± 13.2 |
| ≥ 80 | 32 (5.3) | 1109 (8.7) | 52 (4.2) | 972 (11.6) | 21 (2.2) |
| 50–<80 | 276 (45.5) | 5891 (46.3) | 486 (39.1) | 4351 (52.0) | 418 (43.0) |
| 30–<50 | 256 (42.2) | 4787 (37.7) | 533 (42.9) | 2916 (34.8) | 508 (52.3) |
| 15–<30 | 42 (6.9) | 916 (7.2) | 170 (13.7) | 130 (1.6) | 24 (2.5) |
| < 15 | 0 (0.0) | 8 (0.1) | 1 (0.1) | 2 (0.0) | 1 (0.1) |
| **CHA_2_DS_2_-VASc, mean ± SD** | 4.0 ± 1.3 | 4.1 ± 1.3 | 4.1 ± 1.3 | 3.1 ± 1.1^c^ | 3.1 ± 1.1^c^ |
| **HAS-BLED^b^, mean ± SD** | 2.4 ± 1.0 | 2.7 ± 1.0 | 2.5 ± 1.0 | NA | NA |
| **Edoxaban, 60 mg, %** | 30.2 | 61.0 | 30.4 | 33.5 | 33.6 |
| **Edoxaban, 30 mg, %** | 69.8 | 39.0 | 69.6 | 32.9 | 32.6 |
| **Warfarin, %** | NA | NA | NA | 33.1 | 33.3 |
| **Type of AF** |  |  |  |  |  |
| Paroxysmal | 232 (32.0) | 6293 (47.9) | 618 (44.7) | 2172 (25.6) | 210 (21.5) |
| Persistent | 196 (27.1) | 2291 (17.4) | 306 (22.1) | 1883 (22.2) | 238 (24.4) |
| Long-standing persistent | 117 (16.2) | 1595 (12.1) | 156 (11.3) | NA | NA |
| Permanent | 179 (24.7) | 2959 (22.5) | 303 (21.9) | 4415 (52.1) | 529 (54.1) |
| **Diabetes mellitus** | 195 (26.9) | 3302 (23.9) | 410 (29.6) | 2336 (27.6) | 233 (23.8) |
| **Hypertension** | 533 (73.6) | 10,697 (77.5) | 1050 (75.8) | 7857 (93.7) | 822 (84.0) |
| **Heart failure** | 75 (10.4) | 3090 (22.4) | 205 (14.8) | 3815 (45.0) | 407 (41.6) |
| **COPD** | 40 (5.5) | 811 (5.9) | 100 (7.2) | 858 (10.1) | 69 (7.0) |
| **Peripheral artery disease** | 3 (0.4) | 390 (2.8) | 14 (1.0) | 420 (5.0) | 37 (3.8) |
| **History of ischemic stroke** | 144 (19.9) | 1897 (13.7) | 226 (16.3) | 1336 (15.8)^d^ | 281 (28.7)^d^ |
| **History of major or CRNM bleeding** | 24 (3.3) | 450 (3.3) | 64 (4.6) | NA | NA |
| **History of major bleeding** | 20 (2.8) | 281 (2.0) | 44 (3.2) | NA | NA |
| **Valvular disease** | 31 (4.3) | 1892 (13.7) | 169 (12.2) | 1910 (22.5)^e^ | 131 (13.4)^e^ |

Data presented are mean ± SD for continuous variables and number of patients (percentage) for categorical variables, unless otherwise noted. ^a^Calculated using the Cockcroft-Gault equation. Percentage calculation was done amongst patients with available data. ^b^Modified HAS-BLED excluding labile international normalized ratio. ^c^Datum is CHADS_2_ score. ^d^History of any stroke. ^e^Medical history of valvular heart disease.

AF, atrial fibrillation; CHADS_2,_ congestive heart failure, hypertension, age ≥75 years, and diabetes mellitus; CHA_2_DS_2_-VASc, congestive heart failure, hypertension, age (≥75), diabetes, previous stroke/transient ischemic attack, vascular disease, age (65–74), sex (female); COPD, chronic obstructive pulmonary disease; CrCl, creatinine clearance; CRNM, clinically relevant nonmajor; HAS-BLED, hypertension, abnormal liver/renal function, stroke history, bleeding history or predisposition, elderly, drug/alcohol use; NA, not available; SD, standard deviation.

**Supplementary Table 7.** Annual incidence rate (%) of clinical outcomes in the middle/oldest-old population by regions

|  | **ETNA-AF Korea**  **≥75 years** | **ETNA-AF Global**  **≥75 years** | **ETNA-AF East-Asia**  **≥75 years (2)** | **ENGAGE-AF TIMI 48**  **≥75 years (3)** | **ENGAGE AF-TIMI 48 Asia ≥75 years (3)** |
| --- | --- | --- | --- | --- | --- |
| **Any stroke** | 12 (1.75) | 173 (1.37) | 22 (1.67) | 167 (2.05) | 25 (2.67) |
|  | [1.00; 3.09] | [1.18; 1.59] | [1.10; 2.54] | [1.76; 2.39] | [1.80; 3.95] |
| **Ischemic stroke** | 7 (1.02) | 131 (1.04) | 15 (1.14) | 139 (1.71) | 22 (2.35) |
|  | [0.49; 2.14] | [0.87; 1.23] | [0.69; 1.89] | [1.44; 2.01] | [1.55; 3.57] |
| **Hemorrhagic stroke** | 4 (0.58) | 38 (0.30) | 6 (0.45) | 29 (0.35) | 3 (0.32) |
|  | [0.22; 1.55] | [0.22; 0.41] | [0.20; 1.01] | [0.25; 0.51] | [0.10; 0.98] |
| **SEE** | 0 (0.00) | 12 (0.09) | 1 (0.08) | 17 (0.21) | 2 (0.21) |
|  | [0.00; NE] | [0.05; 0.17] | [0.01; 0.54] | [0.13; 0.33] | [0.05; 0.85] |
| **Any stroke or SEE** | 1 (0.14) | 184 (1.46) | 23 (1.75) | 181 (2.22) | 27 (2.89) |
|  | [0.02; 1.03] | [1.26; 1.69] | [1.16; 2.63] | [1.92; 2.57] | [1.98; 4.21] |
| **Major bleeding** | 6 (0.87) | 188 (1.49) | 25 (1.90) | 376 (4.68) | 42 (4.54) |
|  | [0.39; 1.94] | [1.29; 1.72] | [1.28; 2.81] | [4.23; 5.18] | [3.36; 6.14] |
| ICH | 4 (0.58) | 49 (0.39) | 9 (0.68) | 53 (0.65) | 6 (0.64) |
|  | [0.22; 1.55] | [0.29; 0.51] | [0.35; 1.31] | [0.49; 0.85] | [0.29; 1.41] |
| Major GI bleeding | 2 (0.29) | 107 (0.85) | 13 (0.98) | 178 (2.19) | 12 (1.28) |
|  | [0.07; 1.16] | [0.70; 1.02] | [0.57; 1.69] | [1.89; 2.54] | [0.73; 2.25] |
| **Major or CRNM bleeding** | 9 (1.31) | 470 (3.76) | 43 (3.30) | 1309 (17.60) | 165 (19.36) |
|  | [0.68; 2.52] | [3.44; 4.12] | [2.45; 4.45] | [16.67; 18.57] | [16.62; 22.56] |
| **Major or CRNM GI bleeding** | 3 (0.44) | 349 (2.78) | 25 (1.91) | 226 (2.80) | 31 (3.34) |
|  | [0.14; 1.35] | [2.50; 3.09] | [1.29; 2.82] | [2.46; 3.19] | [2.35; 4.75] |
| **All-cause mortality** | 9 (1.30) | 581 (4.58) | 36 (2.71) | 359 (4.33) | 47 (4.93) |
|  | [0.68; 2.50] | [4.23; 4.97] | [1.96; 3.76] | [3.91; 4.81] | [3.71; 6.57] |
| **CV mortality** | 5 (0.72) | 282 (2.22) | 14 (1.06) | 267 (3.22) | 39 (4.09) |
|  | [0.30; 1.74] | [1.98; 2.50] | [0.63; 1.78] | [2.86; 3.63] | [2.99; 5.60] |
| **Net clinical outcome^a^** | 19 (2.78) | 843 (6.71) | 69 (5.28) | 821 (10.29) | 106 (10.58) |
|  | [1.77; 4.36] | [6.27; 7.18] | [4.17; 6.69] | [9.61; 11.02] | [9.57; 14.02] |

Data are number of patients (percentage) with 95% confidence interval of the incidence rate in square brackets.

**^a^**Net clinical outcome is a composite of ischemic or hemorrhagic stroke, systemic embolic event, major bleeding, and all-cause mortality.

CRNM, clinically relevant nonmajor; CV, cardiovascular; HR, hazard ratio; GI, gastrointestinal; ICH, intracranial hemorrhage; NE, not evaluable; SEE, systemic embolic event.

**Supplementary Figure 1.** Kaplan-Meier curves for the net clinical outcome in different age groups


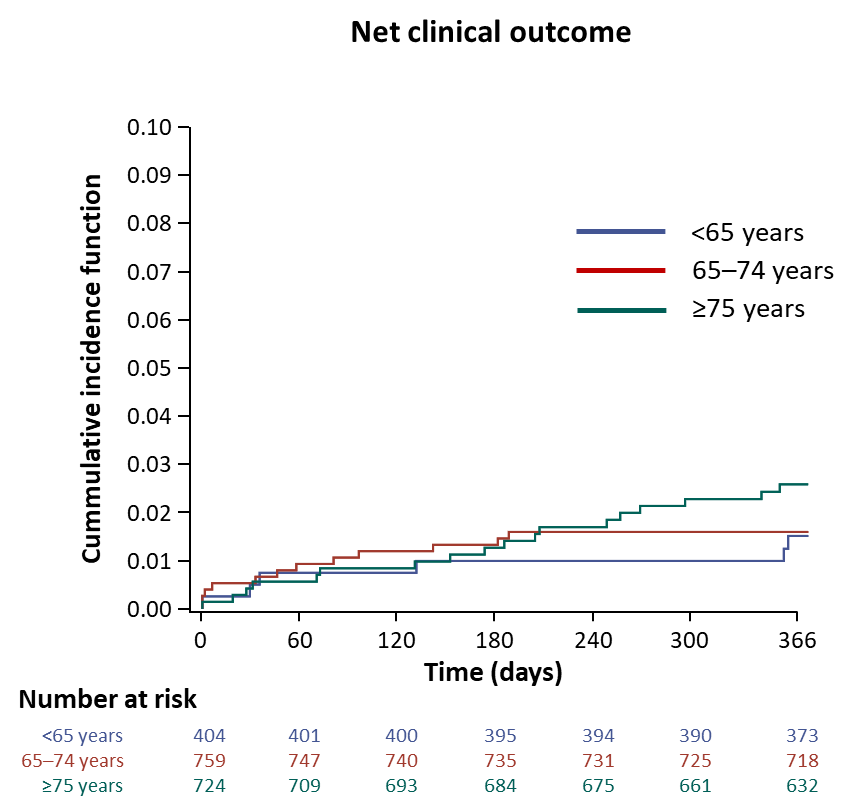
Net clinical outcome is a composite of ischemic or hemorrhagic stroke, systemic embolic event, major bleeding, and all-cause mortality.

**References**

1. Chao TF, Chen SA, Ruff CT, Hamershock RA, Mercuri MF, Antman EM, et al. Clinical outcomes, edoxaban concentration, and anti-factor Xa activity of Asian patients with atrial fibrillation compared with non-Asians in the ENGAGE AF-TIMI 48 trial. Eur Heart J. 2019;40(19):1518-27.

2. K S, editor One-year Clinical Outcomes by Age of 3359 Atrial Fibrillation (AF) Patients on Edoxaban From East Asia Treated in Routine Clinical Practice in the Noninterventional ETNA-AF Program. 14th Asia Pacific Heart Rhythm Society Scientific Session; 2021 November 11-14; China.

3. Kato ET, Giugliano RP, Ruff CT, Koretsune Y, Yamashita T, Kiss RG, et al. Efficacy and Safety of Edoxaban in Elderly Patients With Atrial Fibrillation in the ENGAGE AF-TIMI 48 Trial. J Am Heart Assoc. 2016;5(5):e003432.
